# Genital Microbiota and Outcome of Assisted Reproductive Treatment—A Systematic Review

**DOI:** 10.3390/life12111867

**Published:** 2022-11-12

**Authors:** Rajani Dube, Subhranshu Sekhar Kar

**Affiliations:** 1 Department of Obstetrics and Gynaecology, RAK College of Medical Sciences, Ras Al Khaimah 11172, United Arab Emirates; 2 Department of Paediatrics and Neonatology, RAK College of Medical Sciences, Ras Al Khaimah 11172, United Arab Emirates

**Keywords:** microbiota, assisted reproductive treatment, in vitro fertilization, outcome, infertility

## Abstract

The balance between different bacterial species is essential for optimal vaginal health. Microbiome includes the host genome along with microorganism genomes and incorporates the biotic and abiotic factors, reflecting the habitat as a whole. A significant difference exists in the composition and number of the human microbiota in healthy individuals. About one-tenth of the total body microbiota exists in the urogenital tract and these can be identified by microscopy and culture-based methods, quantitative PCR, next generation and whole genome sequencing. The trend of delaying the planning of pregnancy to a later age nowadays has resulted in magnifying the use of assisted reproductive treatment (ART). Hence, genital microbiota and its impact on fertility has generated immense interest in recent years. In this systematic review, we searched the available evidence on the microbiota of the genital tract in women undergoing ART and studied the outcomes of IVF in different microbial compositions. Despite the inconsistency of the studies, it is evident that vaginal, cervical and endometrial microbiota might play a role in predicting ART outcomes. However, there is no clear evidence yet on whether the diversity, richness, quantity, or composition of species in the maternal genital tract significantly affects the outcomes in ARTs.

## 1. Introduction

Microorganisms are normal inhabitants of the human body and are present in huge numbers at all sites throughout the body [1]. While the microbiome includes the host genome along with microorganism genomes, and incorporates the biotic and abiotic factors, reflecting the habitat as a whole, microbiota refers to the specific microbial taxa that are associated with an environment [2]. The study of microbiota involves the use of advanced molecular techniques [3,4]. Interest in the microbial composition of the female genital tract is in place for more than 50 years now and existed before the first successful in vitro fertilization (IVF) [5]. With the success of the Human Microbiome Project, it was evident that there exists a significant difference in the composition and number of the human microbiota in healthy individuals [6]. About one-tenth of the total body microbiota exists in the urogenital tract [7]. With the advancement of molecular diagnostic techniques and software, microbiota could be found in places earlier considered as sterile, e.g., endometrium, tubes, peritoneum, and even placenta [8].

The study of microbiota of a specific site includes the richness, diversity, identification of species, and relative composition of species. This can be achieved by conventional or newer methods, each with its own advantages and disadvantages.

### 1.1. Microscopy and Culture-Based Methods

These require specific media for bacterial growth and are time-consuming, but non-expensive. While some bacteria cannot be grown in culture, fast-growing bacteria can affect the prevalence studies. The discrimination between the bacteria is based on bacterial morphology and/or biochemical reactions and is highly operator-dependent [9].

### 1.2. Quantitative Polymerase Chain Reaction (qPCR)

It is a fast and affordable method that is well-established for the detection, quantification and typing of different microbial agents. It can identify organisms that are not detected by microscopy or culture and can be used for real-time monitoring of deoxyribonucleic acid (DNA) amplification using fluorescence. In the area of bacterial diagnostics, it can replace culture techniques, especially when rapid and sensitive diagnostic assays are required. However, it does not discriminate between viable and dead organisms and has a limited range compared to next-generation sequencing [10].

### 1.3. Next-Generation Sequencing (NGS)

NGS is a new technology for DNA and RNA sequencing and variant/mutation detection. This technology combines the advantages of unique sequencing chemistries, different sequencing matrices, and bioinformatics technology [11]. The 16S rRNA (ribosomal ribonucleic acid) gene is present in virtually all bacteria, and it has regions of sequence conservation that can be used as targets for PCR, as well as regions of variable sequencing which can be used to differentiate bacteria. There are variable regions (V1-9) that are most commonly used as targets, but the V3/V4 region is used in most of the studies. It is used to identify bacteria, not previously possible with microscopy, culture, or qPCR, and there are databases that are used to classify bacteria based on this targeting [12,13]. This has limitations at the species level.

### 1.4. Whole Genome Sequencing (WGS)

It is a more advanced technique based on massive genome sequencing and is used to reliably differentiate bacteria at species and strain levels. WGS is also not dependent on the availability of predefined databases for comparison and matching [14,15]. However, it is expensive and requires complex analysis.

These techniques are used to know the composition (genus, species, and strains), numbers, and distribution of bacteria. Chao1 index is commonly used to denote the richness of species while Shannon (SDI) or Simpson’s indices are used for a diversity of species. Alpha diversity denotes measures within the sample while beta diversity denotes between samples [6,16,17].

It is generally believed that the balance between different bacterial species is essential for optimal vaginal health [18]. Although *Lactobacilli* are the dominant species in the vagina most often, there are significant variations in normal microbiota between individuals and even in the same individual in different situations such as menstruation, ovulation, and sexual intercourse [19,20]. Therefore, five Community State Types (CST), have been defined in the vagina according to the dominant species: type I is dominated by *L. crispatus*, type II by *L. gasseri*, type III by *L. iners*, type IV by different anaerobic bacteria (such as *Gardnerella* spp., *Prevotella* spp., *Megasphera* spp. or *Sneathia* spp.), and type V by *L. jensenii* [19,20,21,22]. Similar microbiota is believed to be present in the cervix as well [23]. The knowledge about endometrial microbiota is limited, largely because it was earlier thought to be “sterile”. Additionally, more invasive methods are required for sample collection, such as an endometrial biopsy, embryo transfer (ET) catheter tip, or endometrial fluid collection during intrauterine insemination. However, a large number of species of bacteria including *Bacteroides* spp., *Streptococcus* spp., and *Staphylococcus* have been identified, although *Lactobacilli* are the dominant genus in most women [24,25,26,27]. With these findings, a microbial continuum has been recognized to exist throughout the genital tract. The microbiomes were better characterized and the role of healthy microbiota in the reproductive process was realized [27,28]. However, the microbiota in the genital tract can change under the influence of hormones and it has been recently reported that endometrial microbiota can differ between the follicular and luteal phases. Furthermore, the *Lactobacillus* can significantly change with age, and it is lower in women older than 36 years with previous births [29].

The trend of delaying the planning of pregnancy to a later age nowadays has resulted in magnifying the use of assisted reproductive treatment (ART). Hence, genital microbiota and its impact on fertility (and more specifically the success of IVF) has generated immense interest in recent years.

This paper aims to perform a systematic review of the available evidence on the microbiota of the genital tract in women undergoing ART and study the outcomes of IVF in different microbial compositions.

## 2. Materials and Methods

A systematic search of PubMed, Embase, MedRxiv, EBSCO MEDLINE, and Scopus electronic databases was carried out. Medical subject headings (MeSH) and free-text term keywords were used using the following strategy “genital microbiota” OR “genital microbiome” OR “genital microfilm” OR “ genital microflora” OR “vaginal microbiota” OR “vaginal microbiome” OR “vaginal microfilm” OR “vaginal microflora” OR “cervical microbiota” OR “cervical microbiome” OR “cervical microfilm” OR “cervical microflora” OR “endometrial microbiota” OR “ endometrial microbiome” OR “endometrial cervical microfilm” OR “ endometrial microflora” AND “fertility” OR “Infertility” AND “IVF outcome” OR “ART outcome” until 10 June 2022. Thereafter, a manual update was carried out on a weekly basis until 10 July 2022. There were no restrictions on the date of publication. The reference lists of the relevant studies were also manually searched to be included if eligible, especially for the older references.

Selection criteria—The search consisted of only English language articles including case reports, case series, and letters to editors containing case information. After a thorough screening, no randomized clinical trials were found.

Inclusion criteria—Full-text original research articles written in English fulfilling the following criteria (1, 2, and 3 OR 4) were included for review.

1. Women or couples with infertility regardless of the cause or non-pregnant women planning for pregnancy.
2. Studies where the microbial assessment of the genital tract was carried out.
3. Studies where a comparison of the microbiome was available between fertile and infertile women.
4. Studies where a comparison of the microbial flora was available between women with ART/IVF success and failure.

Exclusion criteria—Exclusions consisted of studies where comparisons were not available, duplicated studies, review articles, abstracts, articles in languages other than English, and studies where microbial studies were not carried out. Conference abstracts, editorials, expert opinions, book chapters, and critical appraisals were also excluded.

Both authors (RD and SSK) reviewed all titles and abstracts independently. The potential relevance of the studies to be included for review was agreed on by a discussion. Selected titles and abstracts were further screened between studies to reject overlaps. Full-text copies of the selected papers were obtained and the relevant data regarding study characteristics, evidence of microbiota, and IVF outcomes were extracted by the same two reviewers independently. In the case of individual case reports, if the same patient was included in more than one study with similar characteristics and findings, only the report with a larger number of patients was included. Finally, studies were screened by assessing selection, comparability, and exposure for inclusion into evidence acquisition.

## 3. Results

**Figure 1:**
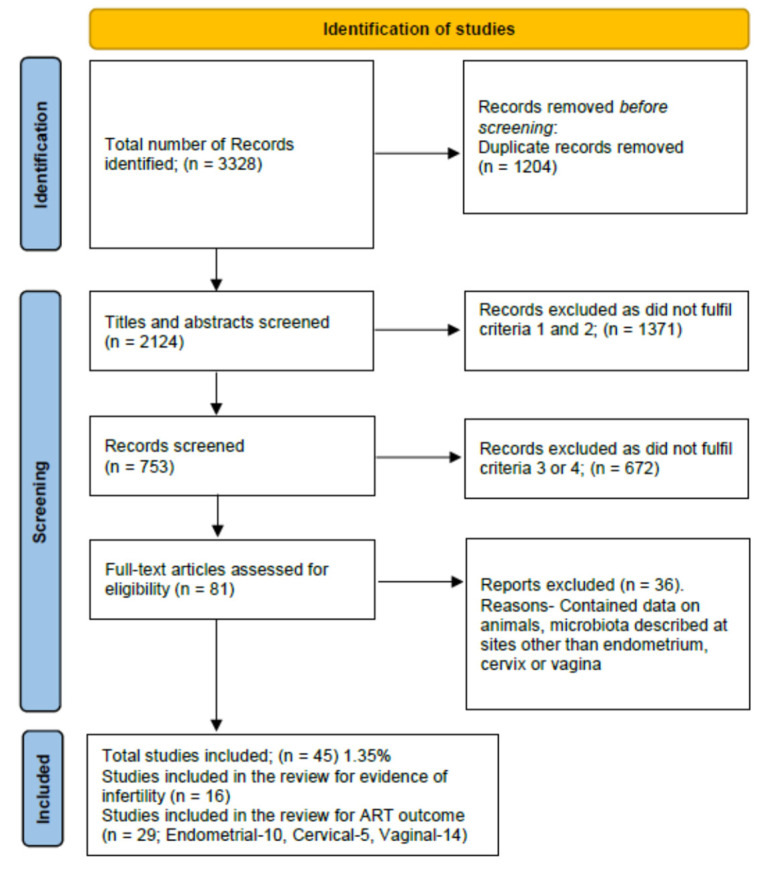
PRISMA flow diagram for inclusion of studies.

After a thorough search, a total of 3328 results were retrieved. All the abstracts and study titles were screened and duplicates were removed. Furthermore, there were 2079 studies excluded as they either did not fit the inclusion criteria (1, 2, 3, 4), were animal studies, microbiota was described at sites other than genital organs, or were trials not yet finished and published. In a manual search of references, two case reports were found and included. Finally, 45 articles were included in the analysis. The Preferred Reporting Items for Systematic Reviews and Meta-Analyses (PRISMA) show the final study inclusions (Figure 1).

In this review, the parameters that were analyzed include the composition of microbiota (richness, diversity, *lactobacilli* predominance, variations in different *lactobacilli* species, and presence of other species) and ART/IVF outcome each in the endometrium, cervix, and vagina. For the purpose of this research, the studies involving samples from ET catheters were considered in the endometrial microbiota group, and catheters used to check cervical blockage just before ET were considered in the cervical microbiota group. 

### 3.1. Genital Microbiota and Association with Infertility

An earlier study by Taylor and Ilesanmi et al. failed to show any growth or Gram-staining of bacteria and pus cells in histological examination and bacteria culturing of endometrial samples in 73 women with primary (n = 12) and secondary infertility (n = 61) [30].

In the study by Moreno et al., it was found that *Lactobacillus, Atopobium, Gardnerella, Prevotella,* and *Sneathia* were found in both endometrium and vaginal samples, but *Lactobacilli* were predominant and present in different percentages between women. *Gardnerella* was present in the vagina but not in the endometrium of certain individuals. It also demonstrated that the endometrial microbiota is different from the vagina and is not a carry-over [31].

In our review, all of the studies showed the absence of *lactobacilli* predominance in the endometrium to be associated with infertility [31,32,33,34,35]. On the other hand, in women with infertility, there was evidence of an infection with *chlamydia, mycobacterium tuberculosis, garderenella* or *E. coli* in the cervix [5,36,37,38,39,40]. In vaginal microbiota, there was an array of microorganisms detected in women with infertility [39,41,42,43] Table 1.

### 3.2. Endometrial Microbiota (EM) and ART Outcome

Franasiak et al. did not find any differences in microbiomes between ongoing (n = 18) and non-ongoing pregnancies (n = 15). In both groups of women, *Flavobacterium* and *Lactobacillus* constituted the majority of the bacterium [45]. An older study in Nigeria revealed no growth of bacteria in endometrial samples of 73 women with (primary and secondary) infertility, although tests for chlamydia were not carried out [30].

Furthermore, in a study by Moreno et al. (2016), it was found that the presence of a non-*Lactobacillus*-dominated microbiota in a receptive endometrium was associated with significant decreases in implantation [60.7% vs. 23.1% (*p* = 0.02)], pregnancy [70.6% vs. 33.3% (*p* = 0.03)], ongoing pregnancy [58.8% vs. 13.3% (*p* = 0.02)], and live birth [58.8% vs. 6.7% (*p* = 0.002)] rates compared to *lactobacilli* dominant microbiota [31].

A recent larger study by Moreno et al. (2022) involving 342 women undergoing IVF demonstrated a pregnancy in 198 women and failure in 144 women. Among the pregnant women, 141 women had live births. *Atopobium, Bifidobacterium*, *Chryseobacterium*, *Gardnerella, Haemophilus*, *Klebsiella*, *Neisseria*, *Staphylococcus*, and *Streptococcus* were seen in women with reproductive failure, whereas the dominant presence of *Lactobacillus* was seen in patients that achieved live births [35].

There were few case reports included in our study. According to a case report, when the endometrial microbiota composition in a woman with recurrent reproductive failure was determined by 16S rRNA gene sequencing, *Gardnerella*, *Atopobium*, and *Bifidobacterium* were detected in the endometrium, and it was found that the *Gardnerella* colonization of endometrium is associated with reproductive failure [46].

Another interesting case report by Moreno et al. showed that in the same patient with miscarriage after IVF pregnancy, non-*Lactobacillus* dominance (5% *Actinobacteria*, 19% *Firmicutes*, and 76% *Proteobacteria* along with *Enterobacteriaceae*, *Streptococcus*, *Pseudomonas, Staphylococcus*) was detected, whereas a *Lactobacillus*-dominated profile (91% of *Firmicutes* and only 9% of *Proteobacteria*) was seen with the successful subsequent pregnancy [47] Table 2.

### 3.3. Cervical Microbiota and Art Outcome

In a study by Salim et al., cervical microbiota was analyzed by bacterial culture in 204 women undergoing ET. It was found that any Gram-negative colonization was associated with no conception. The conception rate was significantly higher among women with sterile cultures (31% of 75) as compared with women in whom any pathogenic microorganism was recovered (16% of 129) [52].

In a study by Fanchini et al. involving samples from the catheter used to check for cervical blockage before ET in 279 women, it was found that 51% of samples were culture-positive. In this study, the most common organism was *Escherichia coli* (64%), followed by *Lactobacillus, Streptococcus* species, *Enterobacteriaceae, anaerobic flora, Enterococcus* species, *Staphylococcus* species, *Haemophilus* species, or the presence of more than one type of microorganism. Clinical and ongoing pregnancy rates as well as implantation rates were significantly lower in the positive culture group than in the negative culture group (24% versus 37%; 17% versus 28%; and 9% versus 16%, respectively). However, it was found that neither the microbial count in the positive culture group nor any particular organism had any significant effect on the success of IVF ET [53] Table 3.

### 3.4. Vaginal Microbiota and IVF Outcome

In a study by J Mangot-Bertrand involving 307 patients treated with IVF, bacterial vaginosis (BV) was found in 9.45%. The embryo implantation rate was decreased in women with BV (36.3% vs. 27.6%, *p* = 0.418), but it was not statistically significant. Furthermore, obstetrical outcomes such as clinical pregnancy rate, early and late miscarriage, premature rupture of membranes, preterm delivery, mode of delivery, and birth weight were not different among women with or without diagnosed BV [62].

In another older study by Moore D et al. in 91 women undergoing IVF-ET, it was found that an increase in the live birth rate was significantly associated with recovery of hydrogen peroxide-producing *Lactobacillus* species from the vagina (*p* = 0.01) [48], as shown in Table 4.

## 4. Discussion

Most of the studies in this review were cohort, case–control or descriptive-type, with few of them having combinations of them.

### 4.1. Association with Infertility

Among the different parts of the genital tract, the lower genital tract and particularly the vagina is long thought to be a bacteria-rich area, and endometrium is considered to be a relatively sterile area. With advances in technology, more studies looked at the normal bacteria flora in different physiological and pathological conditions.

There were a few studies demonstrating the association of endometrial microbiota with infertility. It was suggested by a total of 679 infertile women through five studies that low *lactobacilli* growth in endometrial microbiota was associated with infertility [31,32,33,34,35].

The studies have also shown clear evidence of the association of cervical growth of specific bacteria such as *Chlamydia*,* Gardnerella*,* Mycobacterium tuberculosis*, and* E. coli* with infertility [5,36,37,38,39,40]. It is also evident that a balance of anaerobic-to-aerobic bacteria is favorable for pregnancy and anaerobic predominance is unfavorable [44].

The association of vaginal microbiota with infertility is quite diverse, with most of the studies showing an association of lower *lactobacilli* in the infertility group (in a combined group of 705 patients) [39,41,42,43]. In a recent study involving 478 women planning pregnancy, it was seen that a higher growth of *Lactobacillus* was seen in women who became pregnant, while a higher growth of *Gardnerella* was seen in non-pregnant women. Lower fecundability was associated with a higher abundance of *L. iners*,* Fannyhessea vaginalis*, and a lower abundance of *L. crispatus *and* L. gasseri* [43].

### 4.2. Microbiota and ART Outcomes

#### 4.2.1. Richness and Diversity of Species

There is limited evidence of the correlation of IVF outcomes with the richness and diversity of species in endometrial microbiota. The studies show no difference in women who achieved pregnancy or not after TVF-ET [45]. Diversity did not have an effect on the implantation rate (*p* = 0.85) or miscarriage rate (*p* > 0.32) in women undergoing IVF-ET [31]. The evidence about cervical microbiota is conflicting with Wee et al. showing no effect of richness and diversity on fertility and few other studies including Graspeuntner et al. showing higher diversity in infertile patients [36,39,54,56].

According to the current evidence, a lower richness and diversity of species in the vagina is associated with a higher pregnancy rate after ART [54,55,56]. Studies by Amato et al. and Haahr et al. have shown a lower diversity of species in pregnant women after ART compared to those who failed to achieve pregnancy (*p* = 0.003 with IU. I and *p* = 0.01 with IVF, respectively). Bernabeu et al. found a lower richness of species (*p* = 0.04) in patients who achieved pregnancy after ET without any differences in alpha or beta diversity (*p* = 0.09) [55]. Similarly, Haahr et al. found that a high Shannon index in vaginal microbiota was associated with a lower live birth rate after IVF (*p* = 0.01), and Hyman et al. reported a lower richness and diversity of species (*p* = 0.001) in the group with live births [54,56].

#### 4.2.2. *Lactobacillus* Species and ART Outcomes

*Lactobacillus* Dominance (LD) was reported in almost all studies. While all of the studies included implantation rate or clinical pregnancy rate as the primary outcome, few others included additional parameters such as miscarriage rate, live birth rate, preterm birth, or even birth weight.

(a) Pregnancy Rate

The effect of endometrial LD (LDEM) on pregnancy rate is positive but non-significant in some studies, while there is no correlation in others. Moreno et al. reported higher rates of implantation (61% vs. 23%—*p* = 0.02), pregnancy (70% vs. 33%—*p* = 0.03), and clinical pregnancy (59% vs. 13%—*p* = 0.02) in LDEM compared to patients with non-Lactobacillus dominance (NLDEM), but it was not statistically significant [31]. However, Kyono et al., found no statistically significant differences in pregnancy rates with a LDEM cut-off of ≥90% of the flora, but higher pregnancy rates with a cut-off of 80% [34]. Franasiak et al. also found high loads of *Lactobacillus* spp. and *Flavobacterium* spp., but they observed no relation with pregnancy rates (*p* = 0.75 and *p* = 0.45) [45].

There is conflicting evidence regarding the LD in the vagina and pregnancy rates. However, a number of studies suggest that altered vaginal microbiota may have a negative impact on the outcome of IVF-ET. While more studies show a positive correlation, with a 90% or 80% cut-off favoring pregnancy, other studies show either a negative effect or no effect [34,36,51,54,55,57,60]. In a study by Bernabeu et al. involving 31 women, there was a positive yet insignificant correlation of LD with pregnancy rates (*p* = 0.2) [55]. It may be argued that in women without the required LD, *Bifidobacterium*, a lactic acid-producing bacteria, could have a protective or health-promoting effect in the vagina analogous to that attributed to *Lactobacillus*. However, the study by Wang et al. showed a reduced pregnancy rate in the presence of *Bifidobacterium* [58]. To explain the uncertainties regarding the gross LD and its effect on pregnancy rate, the relative abundance of specific species of *lactobacilli* can be explored in the studies. The studies of the microbiota of the vagina before ET revealed that while women with LD were more likely to get pregnant, lower proportions of *L. crispatus* in the vagina could decrease the chance of pregnancy [50,55]. Furthermore, an imbalance in the vaginal microbiota, as in bacterial vaginosis, may reduce pregnancy rates in IVF patients [65].

(b) Live Birth Rate

Endometrial presence and dominance of the *lactobacillus* species generally are associated with higher live birth rates following ART, but the scenario is unclear about LD in vaginal microbiota. Moreno et al. reported higher live births (59% vs. 6.7%—*p* = 0.02) in patients with an LDEM (load ≥ 90%) compared to patients with NLDEM [31]. In a few other studies, there was no significant correlation between the vaginal load of *Lactobacillus* spp. and a live birth rate, with a *p*-value of 0.42 and 0.2, respectively [54,55]. Similarly, another study also found no effect of vaginal microbiota on live birth rates after IVF treatment [66]. Whether and how vaginal microbiota affects the outcome of ET remains to be further explored.

(c) Miscarriage Rate

Very few studies reported miscarriage rates following ART correlating with EM. Kyono et al., found no statistically significant differences in miscarriage if LDEM is defined as ≥90% of the flora, but lower miscarriage rates if an LDEM cut-off of >80% is used. Other studies found no significant effect of EM on miscarriage rates [31,34,49]. The outcomes were worse when *Gardnerella* spp. or *Streptococcus* spp. were present in the endometrium [31].

#### 4.2.3. Presence of Other Species and ART Outcomes

The endometrial presence of species other than lactobacillus has a conflicting effect on overall ART outcomes, as per the current evidence. Kyono et al. defined dysbiosis as *lactobacilli* of <80% of flora. The dysbiotic flora showed a higher growth of *Atopobium, Gardnerella,* and *Streptococcus* [34,49]. In a recent study involving 99 women undergoing IVF, it was reported that 31.3% (n = 31) had dysbiotic endometrium [49]. Despite this, the individual proportion of these bacteria did not have any impact on the rate of pregnancy or miscarriage rate, and it was similar with or without dysbiosis (52.9% vs. 54.8%) [34,49]. Pregnancy was also detected in the absence of *Lactobacillus* in the endometrium [49]. Furthermore, while *Acinetobacter* spp. and *Pseudomonas* spp. growth was significantly more frequent in the pregnant group (*p* = 0.04 and *p* = 0.004) in one study, the presence of *Gardnerella* was associated with lower pregnancy rates in another [45,51]. *Burkholderia* spp was exclusively present in women with recurrent implantation failure and *Kocuria* in women with unsuccessful IVF-ET [50,51].

The presence of other bacterial species such as *Chlamydia, Gardnerella, Prevotella,* and *Sneathia* in the cervix correlates well with infertility [36]. Among the *Lactobacilli*, the most evidence revolves around *L. crispatus* and *L. iners*. While the former shows a positive correlation with ART outcomes, the latter shows a negative correlation [36,59].

In a recent study by Hao et al. involving 100 women undergoing ET, it was found that 68.8% of clinically pregnant women had microbiota dominated by other bacteria. While low levels of *L. crispatus* were seen in pregnant women using fresh and frozen-thawed ET, low levels of both *L. jensenii* and *L. gasseri* were associated with fresh cycle ET success and high levels of *L. jensenii* and *L. gasseri* were associated with pregnancy in frozen-thawed ET. However, they were not statistically significant [61].

In 2011, Ravel et al. identified five vaginal microbial community state types (CST); four of them (I, II, III, and V) are LD, and CST-IV has increased the abundance of strictly anaerobic bacteria (*Gardnerella, Ureaplasma*) and reduced the presence of *Lactobacillaceae* [2]. Among the *Lactobacilli*, the most evidence revolves around *L. crispatus* and *L. iners*. An interesting study by Vargaro et al. showed that microbiota in women achieving a live birth after ET was similar to those who did not (*p* = 0.43). However, a significantly higher proportion of samples dominated by *L. crispatus* was seen in women achieving a live birth (*p* = 0.021), biochemical pregnancy (*p* = 0.039), and clinical pregnancy (*p* = 0.015) [66]. In a study by Salim et al., the presence of Gram-negative bacteria was associated with no conceptions after ET [52]. It is now known that although *Lactobacilli* are generally considered Gram-positive, some variants of *L. iners* are Gram-negative.

In another study by Selman et al., it was concluded that microbial contamination in the vagina and cervix at the time of ET can be associated with significantly lower pregnancy rates [67]. However, the comparative analysis of the vagina and cervix was not carried out in other studies and the possibility of contamination could not be ruled out [45].

The outcome of IVF was also studied by various authors in relation to the microbiota using specimens other than those that were obtained from the cervix, vagina, or endometrium. Pelzer et al. used follicular fluid samples for the detection of bacteria [19]. It was found that bacteria could be cultured from 99% of samples and the colonization rates did not differ among women with different causes of infertility. Few other studies on seminal fluid microbiota revealed the polymicrobial nature with high alpha diversity indices and phylogenetic diversity but were low in species concentrations [63,68,69]. This way, seminal microbiota differs from the vaginal microbiota. Thus, it is possible that a delicately balanced microbiota resulting from the interaction of the seminal fluid with genital tract microbiota and other environmental factors is optimal for conception and successful pregnancy.

PREDICTIVE MODELS—Koedooder et al. developed a predictive algorithm for failure to achieve pregnancy using vaginal microbiota composition. They described an unfavorable profile using a relative *Lactobacillus* load (35%), the presence of *Gardnerella vaginalis* IST1, or *Proteobacteria* (>28%) of the total bacterial load. Based on the relative abundance of *L. crispatus*, the women with unfavorable profiles (82%) were then stratified into groups with a high and an average chance of pregnancy. This prediction model identified women (18%) with a low chance of achieving pregnancy following fresh ET with an accuracy of 94% (sensitivity, 26%; specificity, 97%). The dominance of *L. crispatus* was an important positive predictor of pregnancy [60]. Similarly, Graspeuntner et al. proposed a model using cervical PCR or culture results to diagnose infectious cases of infertility including sexually transmitted infections, the serologic status of *Chlamydia trachomatis*, and the first 10 taxa more abundant in cervical microbiome sequencing. This model could accurately predict most of the cases of infectious infertility, but not all. However, further evidence is required for its clinical application [36].

Based on the LD detected using NGS, there are certain commercial kits available in the market to assess the endometrial microbiome [70,71]. Furthermore, the likely interventions that can be employed include genital tract lavage and antibiotic treatment in combination with the use of prebiotics or probiotics containing mixtures of different species of *Lactobacilli* such as *L. rhamnosus, Limosilactobacillus fermentum*, *L. acidophilus*, and lactoferrin. However, these are based on limited clinical evidence from observational studies and have restricted utility in clinical applications [72].

Kyono et al. treated NLD patients with probiotics. While the microbiota changed to LD in 100% (n = 9), there was no statistically significant impact on the rate of pregnancy. This may be due to the small sample size [34]. Very recently, a trial by Jepsen et al. involving 74 women referred for IVF revealed that vaginal microbiome was found to improve in a greater proportion in the placebo as opposed to the probiotic group (40% in the placebo group vs. 29% in probiotic). They also observed that there can be a spontaneous improvement of the vaginal microbiome over time, and delaying IVF until optimal conditions arrive can be a choice. However, the sample size was small and there is a need for further research in this area [73].

There were inconsistencies in the studies. The sampling methodology was not always well-defined, even though most studies mentioned it to be before ET. In endometrial samplings, it is practically almost impossible to avoid cervicovaginal contamination in cases where sampling is carried out through the cervix. The laboratory methodology was quite different between studies. Researchers used different kits, targeting different hypervariable regions. Bacterial growth can be very different under physiological or pathological influences as well as during the use of antibiotics. Yet, a large proportion of studies had no data on the timing of sampling concerning the menstrual cycle, the recent use of antibiotics prior to sample collection, universal endometrial receptivity, the exclusion of embryo factors (PGT-a or donation), and the number of embryos transferred.

## 5. Conclusions

Successful pregnancy following ART depends on a complex interplay of variables, with microbiota being one of them. Despite the inconsistency of the studies, it is evident that vaginal, cervical, and endometrial microbiota may play a role in predicting ART outcomes. However, there is no clear evidence yet whether the diversity of species; a relative predominance of *Lactobacillus* spp.; the proportion of different lactobacillus species; or the presence of specific other bacteria in the endometrium, cervix, or vagina may significantly affect the outcomes in ARTs. Further studies are required in this field.

## Figures and Tables

**Table 1:** Genital Microbiota and Association with Infertility.

Genital Tract Area	Findings	Study Population	Author [Reference]
Endometrium	*Lactobacillus* abundance was 2% in CE and 81% in NCE. *L. crispatus* was less abundant in CE. Non-lactobacillus taxa were more abundant in CE, *Anaerococcus* and *Gardnerella* were negatively correlated with relative abundance of *Lactobacillus.*	130 infertile women	Liu et al. [32]
	*Lactobacillus, Atopobium, Clostridium, Gardnerella, Megasphaera, Parvimonas, Prevotella, Sphingomonas,* or *Sneathia genera.*	35 infertile women (2016); 342 infertile women (2022)	Moreno et al. [31,35]
	*Lactobacillus* spp. >90% (n = 33), >70% (n = 53). *Corynebacterium* (n = 40), *Bifidobacterium* (n = 15), *Staphylococcus* spp. (n = 38).	70 infertile women	Tao et al. [33]
	*Lactobacillus* spp. percentage lowest in the IVF patients, followed by non-IVF patients, and highest in healthy volunteers (64% vs. 96% vs. 99.5%). *Lactobacillus* spp. >90% lowest in IVF group (38% vs. 74% vs. 86%).	102 infertile women	Kyono et al. [34]
Cervix	Infertile women had more *Gardnerella* in the cervix.	15 infertile women	Wee et al. [39]
	Higher occurrence of *Mycobacterium tuberculosis* in infertile patients	112 infertile couples	Hok et al. [5]
	Only anaerobic bacteria were found (51% of infertile, 26% of early pregnancy loss, 0% in labor); The largest proportion of patients with both aerobic and anaerobic bacteria was found in the labor group.	47 Women with infertility, early pregnancy loss, and labor	Moberg et al. [44]
	Women examined for infertility had significantly higher levels of anti-chlamydial antibodies.	52 Women with reproductive failure and clinically “inflamed cervixes”, and Infertile women	Koskimies et al. [37]
	*E. coli* growth in cervical samples was associated with infertility.	288 infertile couples	Mishra et al. [40]
	Women with previous *chlamydia* infection (ININF > nININF and FSW); *Lactobacillus*-78.34% in FF, 69% in nININF, 58% in ININF; growth of *Gardnerella, Prevotella,* and *Sneathia* (ININF > nININF > FF).	47 [nININF (n = 26), ININF (n = 21)], FSW (n = 54), FF (n = 89)	Graspeuntner et al. [36]
	*Chlamydia trachomatis* infection 88% in the infertile group vs. 28% in the fertile group.	34 infertile women	Cheong et al. [38]
Vagina	Infertile women had more *Ureaplasma* in the vagina.	15 infertile women	Wee et al. [39]
	*L. iners, L. crispatus,* and *L. gasseri* distinguished idiopathic infertile from other groups. *Fusobacteria* was present in women with bacterial vaginosis but not in women with idiopathic infertility.	96 women with idiopathic infertility, bacterial vaginosis, non-idiopathic infertility, and healthy women	Campisciano et al. et al. [41]
	Detection of *Candida* spp. (27%), *Enterococcus*(23%), *E. coli* (14%) in infertile women. The percentage of *Lactobacillus* was relatively low (4%) and asymptomatic vaginosis was present in 28% of women with infertility.	200 (116 women with Infertility and 84 healthy)	Babu et al. [42]
	Lower fecundability was associated with higher *Actinobacteria, Gardnerella, L. iners, Fannyhessea vaginalis*, and a lower abundance of *L. crispatus* and *L. gasseri*. Higher *Lactobacillales* in the pregnant group.	478 women planning pregnancy	Hong, X. et al. [43]

CE = chronic endometritis, NCE = non-chronic endometritis, *L. crispatus* = *Lactobacillus crispatus*, *L. iners* = *Lactobacillus iners*, *E. coli* = *Escherichia coli*, ININF = infectious infertility, nININF = non-infectious infertility, FSW = female sex workers, FF = Fertile females.

**Table 2:** Effect of Endometrial Microbiota on ART Outcome.

Parameter	Outcome * [Reference]	No Effect Author [Reference]	Positive Correlation Author [Reference]	Negative Correlation Author [Reference]
High richness of species	On-going pregnancy	Franasiak et al. [27] Moreno et al. [31]		
High diversity		Franasiak et al. [27] Moreno et al. [31]		
High % of *Lactobacillus* sp.**	On-going pregnancy [33,34,35] Live birth rate [33,35,48] Miscarriage [34]	Franasiak et al. [27] Kyono et al. [34] # Hashimoto et al. [49] $	Moreno et al. [31] (# or $) Kyono et al. [34] $ Moore et al. [48]	Tao et al. [33] # Riganelli et al. [50]
*Bifidobacterium* sp. **	On-going pregnancy [34]	Kyono et al. [34] # Hashimoto et al. [49] $	Kyono et al. [34] (≥80%)	
*Gardnerella* sp.		Hashimoto et al. [49]		Moreno et al. [35] Kitaya et al. [51]
*Streptococcus* sp.		Hashimoto et al. [49]		Moreno et al. [35]
*Atopobium* sp.		Hashimoto et al. [49]		
*Flavobacterium* sp.		Franasiak et al. [27]		
*Pseudomonas* sp.			Franasiak et al. [27]	
*Acinetobacter* sp.			Franasiak et al. [27]	
*Burkholderia* sp.	RIF [51]			Kitaya et al. [51]
*Kocuria dechangensis*				Riganelli et al. [50]

sp. = Species, RIF = Recurrent implantation failure. * All studies included pregnancy rate/implantation rate/chemical pregnancy. Thus, any additional outcomes are mentioned here. ** Cut-off ≥ 90% = #, cut-off value ≥ 80% = $.

**Table 3:** Effect of Cervical Microbiota on ART Outcome.

Parameter	Outcome * [Reference]	No Effect Author [Reference]	Positive Correlation Author [Reference]	Negative Correlation Author [Reference]	Comments
High richness of species				Hyman et al. [54] Bernabeu et al. [55]	
High diversity				Graspeuntner et al. [36] Hyman et al. [54] Haahr et al. [56] Amato et al. [57]	
High % of *Lactobacillus* sp. **		Hyman et al. [54] Bernabeu et al. [55] Kyono et al. [34] # Wang et al. [58]	Graspeuntner et al. [36] (infectious) Kyono et al. [34] $		Different cut-off values correlate differently
*L. crispatus* (CST 1)		Haahr et al. [56]	Graspeuntner et al. [36] Villani et al. [59]	Koedooder et al. [60] Hao et al. [61]	Low levels in fresh ET and frozen-thawed ET favored pregnancy
*L. gasseri* (CST 2)		Haahr et al. [56]	Hao et al. [61]	Graspeuntner et al. [36] Hao et al. [61]	Low levels in fresh ET and high levels in frozen-thawed ET favored pregnancy
*L. iners* (CST 3)		Graspeuntner et al. [36]		Villani et al. [59]	
*L. jensenii* (CST 5)		Haahr et al. [56]	Hao et al. [61]	Koedooder et al. [60] Hao et al. [61]	Low levels in fresh ET and high levels in frozen-thawed ET favored pregnancy
CST 4 (diverse bacteria) Presence of other species—No effect—Haahr et al. [56]					
*Bifidobacterium* sp.			Villani et al. [59]		
*Gardnerella* sp.		Kitaya et al. [51] Bernabeu et al. [55]		Koedooder et al. [60] Wee et al. [39] Graspeuntner et al. [36]	
*Streptococcus* sp.		Bernabeu et al. [55]		Wang et al. [58]	
*Atopobium* sp.				Villani et al. [59]	
*Sneathia* sp.				Graspeuntner et al. [36]	
*Ureaplasma parvum*		Bernabeu et al. [55]			
*Prevotella* sp.				Graspeuntner et al. [36]	
*Clostridium*		Bernabeu et al. [55]			
*Proteobacteria*			Villani et al. [59]	Koedooder et al. [60]	
*Solanum torvum*				Wang et al. [58]	
*Fusobacterium*				Wang et al. [58]	
*Yersinia*			Villani et al. [59]		

sp. = Species. * All studies included pregnancy rate/implantation rate/chemical pregnancy. Thus, any additional outcomes are mentioned here. ** Cut-off ≥ 90% = #, cut-off value ≥ 80% = $.

**Table 4:** Effect of Vaginal Microbiota on ART Outcome.

Parameter	Outcome * [Reference]	No Effect Author [Reference]	Positive Correlation Author [Reference]	Negative Correlation Author [Reference]	Comments
High richness of species		Wee et al. [39]		Campisciano et al. [41] Hyman et al. [54] Bernabeu et al. [55]	
High diversity		Wee et al. [39] Amato et al. [57] Bernabeu et al. [55]		Campisciano et al. [41] Graspeuntner et al. [36] Hyman et al. [54] Haahr et al. [56] Amato et al. [57] Bernabeu et al. [55]	While alpha-diversity has unfavorable outcomes, betadiversity has no effect
High % of *Lactobacillus* sp. **	RIF [51]	Hyman et al. [54]	Graspeuntner et al. [36] (infectious) Kyono et al. [34] ($ or #) Bernabeu et al. [55] (*p* = 0.2) Koedooder et al. [60]	Kitaya et al. [51] #	Low load of lactobacilli associated with low pregnancy rate
*L. crispatus* (CST 1)	Live birth, miscarriage [63]	Haahr et al. [56]	Campisciano et al. [41] Graspeuntner et al. [36] Koedooder et al. [60] Riganelli et al. [50]	Koedooder et al. [60] Okwelogu et al. [63]	<60% has positive and >60% has negative correlation [60]
*L. gasseri* (CST 2)		Haahr et al. [56]	Okwelogu et al. [63] B. Lledo et al. [64]	Riganelli et al. [50] Campisciano et al. [41] Graspeuntner et al. [36]	
*L. iners* (CST 3)	Live birth, miscarriage [63] PTB [64]	Graspeuntner et al. [36]	Riganelli et al. [50]	Wang et al. [58] Okwelogu et al. [63] B. Lledo et al. [64]	
*L. jensenii* (CST 5)		Haahr et al. [56]		Koedooder et al. [60]	
CST 4 (diverse bacteria) Presence of other species—No effect—Haahr et al. [56]					
*Bifidobacterium* sp.		Amato et al. [57]		Amato et al. [57] Wang et al. [58]	
*Gardnerella* sp.				Koedooder et al. [60] Campisciano et al. [41] Wee et al. [39] Graspeuntner et al. [36] Bernabeu et al. [55] (*p* = 0.11) Riganelli et al. [50]	IS-pro type 1 (IST1) is associated with low pregnancy rate
*Staphyllococcus* sp.				Campisciano et al. [41]	
*Streptococcus* sp.		Bernabeu et al. [55]		Riganelli et al. [50]	
*Atopobium* sp.				Campisciano et al. [41]	
*Sneathia* sp.				Graspeuntner et al. [36]	
*Ureaplasma parvum*		Bernabeu et al. [55]		Campisciano et al. [41] Wee et al. [39]	
*Burkholderia* sp.		Kitaya et al. [51]			
*Prevotella* sp.				Campisciano et al. [41] Graspeuntner et al. [36] Wang et al. [58]	
*Veillonella* sp.				Campisciano et al. [41]	
*Closteridium*		Bernabeu et al. [55]			
*Proteobacteria*				Koedooder et al. [60]	
Bacteroides	Live birth, miscarriage [63]			Okwelogu et al. [63]	

sp. = Species, RIF = Recurrent implantation failure, PTB = Preterm birth, LBR = Live birth rate. * All studies included pregnancy rate/implantation rate/chemical pregnancy. Thus, any additional outcome is mentioned here. ** Cut-off ≥ 90% = #, cut-off value ≥ 80% = $.

## Data Availability

No new data were created or analyzed in this study. Data sharing is not applicable to this article.
